# Overweight and Obesity Is Associated with Higher Risk of Perceived Stress and Poor Sleep Quality in Young Adults

**DOI:** 10.3390/medicina60060983

**Published:** 2024-06-14

**Authors:** Antonios Dakanalis, Gavriela Voulgaridou, Olga Alexatou, Sousana K. Papadopoulou, Constantina Jacovides, Agathi Pritsa, Maria Chrysafi, Elena Papacosta, Maria G. Kapetanou, Gerasimos Tsourouflis, Marina Antonopoulou, Maria Mitsiou, Georgios Antasouras, Constantinos Giaginis

**Affiliations:** 1 Department of Mental Health, Fondazione IRCCS San Gerardo dei Tintori, 20900 Monza, Italy; 2 School of Medicine and Surgery, University of Milano-Bicocca, 20900 Monza, Italy; 3 Department of Nutritional Sciences and Dietetics, Faculty of Health Sciences, International Hellenic University, 57001 Thessaloniki, Greece; gabivoulg@gmail.com (G.V.); souzpapa@gmail.com (S.K.P.); con.jacovides@gmail.com (C.J.); agpritsa@ihu.gr (A.P.); 4 Department of Food Science and Nutrition, School of the Environment, University of the Aegean, 81400 Lemnos, Greece; fnsd23003@fns.aegean.gr (O.A.); m.chrisafi3@gmail.com (M.C.); mariakaptain@yahoo.gr (M.G.K.); mk.antonopoulou@yahoo.com (M.A.); g.antasouras@gmail.com (G.A.); 5 Department of Physical Education and Sport Sciences, School of Education and Social Sciences, Frederick University, 3080 Limassol, Cyprus; aero.pe@frederick.ac.cy; 6 Second Department of Propedeutic Surgery, Medical School, University of Athens, 11527 Athens, Greece; gtsourouflis@med.uoa.gr; 7 Department of Physiotherapy, School of Health Sciences, International Hellenic University, 57001 Thessaloniki, Greece; mitsioumaria@gmail.com

**Keywords:** overweight, obesity, stress, sleep quality, young adults, academic performance, body mass index, university students

## Abstract

*Background and Objectives*: Overweight and obesity are growing public health challenges, particularly concerning young adults. University life presents a unique set of stressors that may influence weight management alongside sleep quality. In this cross-sectional study, we aimed to investigate the association between overweight or obesity, stress, and sleep quality in a large sample of Greek university students. *Materials and Methods*: The study recruited 2116 active students from across various Greek universities. Participants completed questionnaires on sociodemographics, academic performance, and physical activity levels using the International Physical Activity Questionnaire (IPAQ). Stress and sleep quality were assessed using the Perceived Stress Scale (PSS) and the Pittsburgh Sleep Quality Index (PSQI), respectively. Body weight and height were directly measured to calculate Body Mass Index (BMI). *Results*: Our analysis of 2116 Greek university students revealed significant associations between various factors and overweight/obesity. Compared to their rural counterparts, young adults in urban areas had an 88% higher prevalence of overweight/obesity (*p* = 0.0056). Regular smokers were twice as likely to be overweight or obese (*p* = 0.0012). Notably, those with low physical activity levels displayed a more than two-fold increased risk (*p* = 0.0008) compared to those with moderate or high activity levels. Similarly, students with moderate or high perceived stress levels had a more than two-fold prevalence of overweight/obesity compared to those with low stress (*p* = 0.0005). Inadequate sleep quality was also associated with an 86% higher risk of overweight/obesity (*p* = 0.0007). Interestingly, good academic performance showed a 57% greater prevalence of overweight/obesity compared to very good/excellent performance (*p* = 0.0103). *Conclusions*: Our findings reveal that perceived stress and poor sleep quality are significant risk factors for overweight and obesity in this young adult population.

## 1. Introduction

Obesity and overweight represent significant global health challenges, with prevalence rates continuing to rise in recent decades. Between 1976 and 2018, obesity increased by 27.1% among ages 18–25 years [1]. Overweight and obesity are not only associated with serious health problems like cardiovascular diseases and diabetes mellitus type 2 but also with emerging evidence of negative social and academic consequences [2,3,4,5]. Beyond the immediate health risks, obesity in young adults can lead to an increased risk of developing chronic diseases such as certain cancers, musculoskeletal problems, and respiratory issues later in life [6]. The economic burden of young adult obesity on healthcare systems is also a growing concern [7]. While biological and genetic factors appear to play a considerable role in BMI [8], lifestyle choices such as dietary habits, physical activity levels, and a sedentary lifestyle significantly influence weight management in young adults [9].

Mental health disorders have become a growing public health concern, particularly among young adults enrolled both in undergraduate and postgraduate university programs worldwide [10]. The transition to university often brings significant academic and social changes, leading to a potentially stressful period for young adults. This rise in mental health issues underscores the need for a deeper understanding of the factors influencing the well-being status of young adults. University life presents a unique set of challenges for students, including, among others, academic pressures, social anxieties, and stress [11]. Stress is a physiological and behavioral response to stimuli during which the brain can exert a crucial role in interpreting what is perceived as stressful [12]. Chronic stress can also exert detrimental effects on health and well-being, and it is linked to negative health outcomes. In addition, anxiety can lead to depression, and both of these disorders are interrelated and could result in a higher risk of comorbidities [13,14]. Sleep deprivation impairs students’ cognitive abilities like attention and effort allocation during tasks, which may negatively affect students’ cognitive performance [15]. Also, poor sleep quality is implicated in a host of adverse health effects, including emotional dysregulation and heightened susceptibility to chronic diseases [16,17]. Based on the above evidence, anxiety may be emerging as the most prevalent and serious issue among college students, particularly affecting female students [18].

Evidence shows that overweight/obesity is associated with anxiety and poor sleep quality. Notably, anxiety and anxiety-related behaviors have been associated with recurrent episodes of consumption of high amounts of foods in young and older adults, especially foods including high amounts of sugar and fat [19,20]. Existing research has supported substantial evidence that there is a link between stress, sleep quality, and body weight management in young adults. Moreover, several previous studies have shown that young adults experiencing chronic stress and poor sleep are more likely to be affected by overweight or obesity [19,20]. Furthermore, it is currently well-recognized that obesity is frequently associated with sleep disorders such as obstructive sleep apnea (OSA), which can severely compromise sleep quality [21,22]. Sleep restriction, which is positively correlated with anxiety, can also increase food consumption, consequently leading to abdominal visceral obesity, which is a more effective risk factor for morbidity and mortality [23]. However, further research is urgently needed to understand the specific impacts of all these factors on young adults struggling with body weight management.

Currently, there is substantial evidence that the Mediterranean Diet (MD) is one of the most well-recognized healthy dietary patterns worldwide, especially in young populations [24]. The MD was born in the Mediterranean basin and it was first quite poor and simple based mainly on the products that grew almost spontaneously along the shores of the Mediterranean, such as olives, grapes, and wheat, which were long cultivated in the Mediterranean basin [25]. The MD is essentially the dietary pattern that was adopted in ancient times by the populations living along the Mediterranean coast, mainly Greek and Roman populations [25]. Thus, it is reasonable that Greece is distinguished by its national dietary characteristics and that the Greek population may have a higher adherence to traditional and more healthy meals related to MD compared to other populations that live further away from the Mediterranean basin. However, a study assessing MD adherence in young Greek university students compared with a non-Mediterranean (Dutch) population did not confirm the above belief [26]. In fact, this study found that students at both sites showed average adherence to the MD, with slightly higher scores in the Dutch sample compared to the Greek one [26]. The above findings indicate that the MD has been transmitted to non-Mediterranean populations, possibly as a result of its declared health benefits. Conversely, it is alarming that only an average adherence score was demonstrated by young Greek students [26]. Moreover, another comparative study was performed on 663 young Croatian university students to evaluate their diet quality assessed by the Mediterranean Dietary Quality Index (M-DQI) and explore differences between those living in continental and Mediterranean regions [27]. This study found that students’ MD adherence was the same for both regions, presenting a low diet quality [27].

Several studies have investigated the association between BMI and factors such as stress and sleep quality [19,28,29,30]. However, to the best of our knowledge, no study has examined this relationship in a large sample of young adults who are academic students in Greece. By focusing on Greek university students, we can gain valuable insights into the specific challenges faced by this young adult population within the Greek context. Notably, based on previous evidence, which showed that student nutritional habits and lifestyle are rather similar and common amongst most European countries, the results of the present study may be representative of at least European countries [26,27]. In view of the above consideration, as the first step, this study aims to investigate the association between stress, sleep quality, and overweight/obesity to determine whether chronic stress and poor sleep quality are significant risk factors for overweight and obesity in Greek university students. As the second step, this study aims to explore the relationship between overweight or obesity and other relevant factors that may influence overall well-being in young adults, such as socio-demographic and economic background, physical activity levels, and academic performance.

## 2. Methods

### 2.1. Study Population

**Figure 1:**
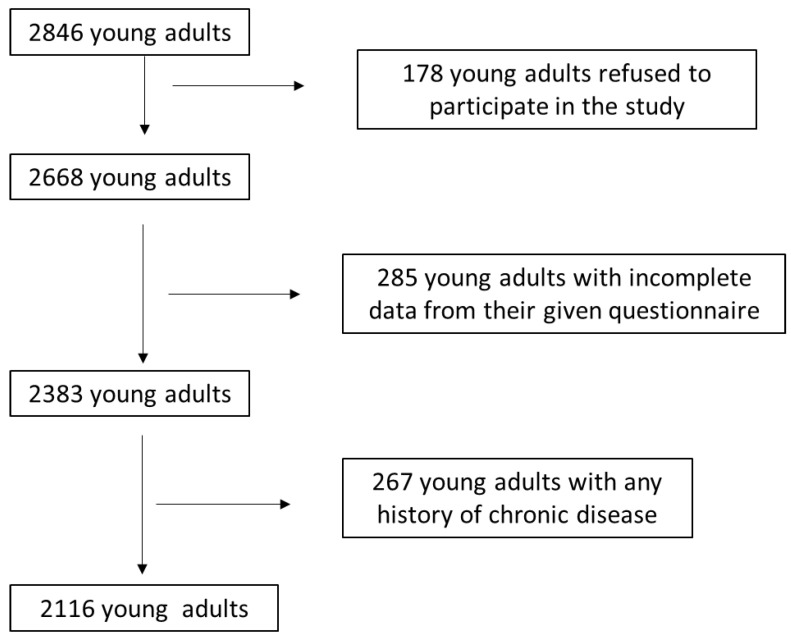
Flow chart diagram of the study population assignment.

The present study primarily included 3075 young adults assigned to 8 geographically various Greek regions, both rural and urban (Athens, Thessaloniki, Larissa, Kavala, Korinthos, Alexandroupolis, and South and North Aegean). We exclusively enrolled young adults who are active students in any Greek university. Enrollment in the study took place between March 2021 and March 2024. Figure 1 shows the flow chart diagram of the participant assignment in detail. By using appropriate exclusion and inclusion criteria, 2116 active university students were finally involved, leading to a final response rate of 68.8%.

The Ethics Committee of the University of the Aegean authorized this study (ethics approve code: no 21/11.10.2017, approval date: 11 October 2017) in accordance with the World Health Organization (52nd WMA General Assembly, Edinburgh, Scotland, 2000). All of the enrolled young adults’ data were confidential, none of the assigned young adults had any history of chronic disorder, such as cardiovascular diseases, metabolic disorders, mental disease, or cancer, and all participants were informed concerning the purposes of the present study by signing a permission document. Sample size computation was conducted utilizing PS: Power and Sample Size estimator software 3.1. Randomization was conducted using a series of random binary numbers (i.e., 100100101, in which 0 represented enrollment and 1 represented non-enrollment in the study). The computation of the power of our sample size showed a power of 88.1%.

### 2.2. Assessment of Sociodemographic and Anthropometric Factors

Appropriate questionnaires were applied to collect the socio-demographic parameters of the enrolled young adults such as age, gender, nationality, type of residence, family economic status, living status, parents’ marital status, smoking habits, and employment status. Moreover, as all the enrolled young adults were active students in any Greek university, their academic performance as well as the type of their studies were recorded. All the socio-demographic characteristics were collected through one-to-one interviews between participating young adults and qualified personnel to lower recall bias. Family economic status was categorized according to the annual family income as low for family annual income ≤ 10,000 €, medium for annual income >10,000 € and ≤20,000 €, and high for annual income > 20,000 €. Academic performance was classified based on the present mean degree of lessons received at the time of study as low for degrees between 5.0 and 6.49, medium for degrees between 6.5 and 8.49, and high for degrees between 8.5 and 10.0 based on the formal criteria of the Minister of Education of Greece.

Body weights and heights of the assigned young adults were measured data recorded at the time of study to calculate Body Mass Index (BMI). Young adults’ weights were measured using the Seca scale (Seca, Hanover, MD, USA) with no shoes to the closest 100 g, and height was measured using a portable stadiometer (GIMA Stadiometer 27335) without shoes to the closest 0.1 cm [31,32]. The WHO guidelines were used to classify the enrolled young adults as normal weight, overweight, or obese at the first week of gestation [31,32].

### 2.3. Assessment of Lifestyle Factors

Physical activity levels were assessed using the International Physical Activity Questionnaire (IPAQ) in which subjects mention how much exercise they did in a typical week. This self-administered questionnaire, used worldwide, assesses the overall physical activity over the last seven days to categorize it as low, moderate, or high [33]. IPAQ instruments have been tested in both developed and developing countries and demonstrated good reliability and acceptable validity properties, at least as good as other self-answered PAQs. Briefly, the purpose of IPAQ-Gr is to sum up vigorous, moderate, and walking PAs over the previous seven-day period and generate a total physical activity score (PA score), expressed in MET-minutes per week (MET.min.wk-1) [33].

The Perceived Stress Scale (PSS) was used to assess the stress of the enrolled young adults [34]. This is a 10-item questionnaire designed to evaluate the self-reported amount of stress in the participants by assessing thoughts and feelings during the last few months. Items were designed to evaluate how unpredictable, uncontrollable, and overloaded the enrolled respondents find their lives. The validity of the PSS has been shown in recent research [35]. The participants had to indicate how often they experienced a certain feeling in the previous month. Each question is scored from 0 (never) to 5 (very often). The total score from the sum of questions may vary from 0 to 40, and perceived stress is higher as scores increase. The PSS is classified into three groups, which are low perceived stress (low PSS; 0–13), medium perceived stress (medium PSS; 14–26), and high perceived stress (high PSS; 27–40) [35].

Sleep quality was assessed using the Pittsburgh Sleep Quality Index (PSQI), which consists of 19 items that are rated on a four-point scale (0–3) and grouped into seven components (sleep quality, sleep latency, sleep duration, habitual sleep efficiency, sleep disturbance, use of sleeping medications, and daytime dysfunction). The item scores in each component were summed and converted to component scores ranging from 0 (better) to 3 (worse) based on guidelines [36]. Total PSQI scores were calculated as the summation of seven component scores ranging from zero to 21, where a higher score indicates a worse condition. A total global PSQI score of <5 is indicative of adequate sleep quality guidelines [36]. All the above questionnaires were completed by trained physicians (e.g., medical and nursing personnel) and nutritionists and dietitians during one-to-one interviews with the enrolled young adults.

### 2.4. Statistical Analysis

The continuous variables that followed normal distributions were treated by Student’s *t*-test. The normal distribution was tested using the Kolmogorov–Smirnov test. Chi-square was applied for categorical variables. Mean value ± Standard Deviation (SD) was used to express quantitative variables that followed a normal distribution. The qualitative variables are stated as absolute or relative occurrences. Multivariate binary logistic regression analysis was used to assess if young adults’ overweight/obesity could be independently associated with socio-demographic, anthropometry, and lifestyle characteristics, including perceived stress and sleep quality, by adjusting for potential confounders. The Statistica 10.0 software, Europe was used for the statistical analysis (Informer Technologies, Inc., Hamburg, Germany).

## 3. Results

### 3.1. Descriptive Statistics of the Study Population

The descriptive statistics of the enrolled study population are included in Table 1. Overall, 2116 young adults with a mean age of 21.4 ± 2.3 years old participated in the present study. In total, 52% of the enrolled young adults were male and 79% of them had Greek nationality. Furthermore, 61.1% of the enrolled young adults lived in urban regions of Greece and 37.9% lived in rural regions. Moreover, 41.6% of the assigned young adults had low economic status, 39.2% of them had medium economic status, and only 19.1% had high economic status. Regarding living conditions, 47.6% of the enrolled young adults lived with their family and the remaining 52.3% lived alone. Lastly, 63.0% of the enrolled young adults had divorced parents and 38.0% of them were regular smokers.

In addition, 45% of the assigned young adults have taken a course in biomedical studies such as medical, nursing, pharmaceutical, or nutritional studies. Moreover, 38.2% of the assigned young adults had good academic performance, 45.4% of them had very good academic performance, and only 16.4% of them had excellent academic performance. In addition, 23.1% of the enrolled young adults were employed. Finally, 15.9% of the enrolled young adults were classified as overweight and 8.6 as obese.

Concerning the lifestyle characteristics of the study population, 48.4% of the enrolled young adults had low physical activity, 33.8% of them exhibited medium activity, and only 17.8% of them had high physical activity. Moreover, 45.6% of the assigned young adults showed low perceived stress, 34.9% presented medium, and 19.5% exhibited high perceived stress. Lastly, 37.5% of the enrolled young adults were shown to have inadequate sleep quality.

### 3.2. Association of BMI Status with Sociodemographic, Anthropometric and Lifestyle Factors

In cross-tabulation, male young adults were more frequently observed to be overweight or obese than female young adults (Table 2, *p* < 0.0001). Greek young adults were marginally less overweight or obese compared to those of different nationalities (Table 2, *p* = 0.0491). Young adults living in urban regions were more frequently observed to be overweight or obese than those living in rural regions (Table 2, *p* < 0.0001). A reverse association between overweight/obesity and economic status was noted (Table 2, *p* = 0.0152).

Young adults living alone were more often overweight or obese than those living with their family (Table 2, *p* = 0.0005). Young adults whose parents had been divorced were more frequently affected by overweight or obesity compared to the young adults whose parents had not been divorced (Table 2, *p* = 0.0309). Overweight and obese young adults were significantly more often regular smokers compared to normal-weight young adults (Table 2, *p* < 0.0001). In addition, overweight and obese young adults showed significantly lower academic performance compared to normal-weight young adults (Table 2, *p* = 0.0002). Participants’ age, employment status, and type of studies were not associated with BMI status (*p* > 0.05).

Concerning lifestyle characteristics, overweight and obese young adults showed significantly lower physical activity levels than their normal-weight peers (Table 2, *p* < 0.0001). Young adults presenting medium or high perceived stress were more likely to be overweight or obese than those with low perceived stress (Table 2, *p* < 0.0001). In addition, overweight and obese young adults showed significantly lower sleep quality compared to normal-weight young adults (Table 2, *p* < 0.0001).

### 3.3. Multivariate Regression Analysis for BMI Status of the Study Population

In multivariate binary logistic regression analysis, young adults’ overweight/obesity was independently associated with type of residence, smoking status, physical activity, perceived stress, and sleep quality after adjustment for multiple potential confounding factors (Table 3, *p* < 0.05). Specifically, young adults living in urban regions showed an 88% significantly higher prevalence of overweight or obesity compared to those living in rural regions (Table 3, *p* = 0.0056). Moreover, regular-smoker young adults had a two-fold higher probability of being overweight or obese than never-smoker young adults (Table 3, *p* = 0.0012). All the other socio-demographic characteristics were non-significant in the multivariate analysis (Table 3, *p* ≥ 0.05).

As far as the lifestyle characteristics were concerned, young adults with low physical activity levels showed a more than two-fold higher likelihood of being overweight or obese compared to those with medium or high physical activity levels (Table 3, *p* = 0.0008). In addition, young adults presenting moderate or high perceived stress had a more than two-fold higher prevalence of being overweight or obese than those presenting low perceived stress (Table 3, *p* = 0.0005). Accordingly, young adults with inadequate sleep quality exhibited an 86% higher incidence of being overweight or obese compared to those with adequate sleep quality (Table 3, *p* = 0.0007). Lastly, young adults with good academic performance showed a 57% greater prevalence of being overweight or obese compared to those with very good/excellent academic performance (Table 3, *p* = 0.0103).

## 4. Discussion

The detrimental effects of overweight and obesity are well-documented, impacting both physical and social aspects of life. Effective body weight management depends on adopting and maintaining healthy behaviors, which are demonstrably susceptible to environmental influences. The lives of academic students often present challenges to healthy habits due to factors like academic pressures, peer influences, and media portrayals, which can also contribute to increased stress. These factors can lead to changes in physical activity levels and sleep duration and quality, potentially increasing the risk of body weight-related disorders like overweight and obesity in university students [37]. Therefore, this study aimed to investigate the association between overweight or obesity and anxiety, poor sleep quality, and stress among young adults, particularly university students. Our findings demonstrated a positive association between increased body mass index (BMI) and both perceived stress and poor sleep quality. Additionally, we observed that living area, tobacco use, physical activity level, and academic performance were also related to BMI.

Our findings reveal a higher prevalence of overweight or obesity among male young adults compared to their female counterparts. This observation does not align with the existing literature, which indicates a higher overall obesity prevalence in females (40%) compared to males (35%) according to the National Health and Nutrition Examination Survey (NHANES) spanning from 2005 to 2014 [38]. This discrepancy highlights the need to examine gender disparities within specific populations. Moreover, this discrepancy may be ascribed to the fact that NHANES includes a study population of adults with a mean age of 48.4 years old, whereas our study population includes younger adults with a mean age of 21.4 years old. NHANES also includes a more multinational study population, whereas, in our study, 79.0% of the enrolled young adults were Greeks. In Italy, males have a higher obesity rate (51%) compared to females (34%) [39]. Understanding these variations across countries requires a multi-factorial approach that considers not only biological and behavioral factors but also socio-economic influences. Our findings support the above notion, as lower family economic status appears to be associated with an increased prevalence of overweight and obesity. The established link between poverty and obesity can be explained by several factors, including limited access to healthy and affordable food options, decreased opportunities for physical activity due to safety concerns or resource limitations, and potentially lower parental monitoring of dietary choices [40].

Rapid industrialization and economic growth in developing countries often trigger a mass exodus from rural areas to cities [41]. This shift in lifestyle is well-documented as a contributing factor to the rise in obesity rates [42]. Consistent with this trend, our study found an 88% increase in the prevalence of overweight or obesity among adults residing in urban regions compared to their rural counterparts. Furthermore, research among Indian young adults supports this association, demonstrating higher BMI and waist-to-hip ratio (WHR) (an effective marker of abdominal obesity) in both urban men and women compared to their rural peers [43]. Interestingly, one might expect globalization to blur the socioeconomic divide between rural and urban areas, potentially leading to a smaller disparity in obesity prevalence. However, our findings suggest that this might not be the case for all populations, highlighting the need for further investigation across diverse socioeconomic contexts.

Studies investigating the link between smoking and BMI have yielded inconsistent results, with some reporting no correlation [44,45], while others showed a higher prevalence of obesity in smokers [46,47]. Our own data align with the above findings, suggesting a link. Young adults who reported regular smoking were twice as likely to be overweight or obese compared to their never-smoking counterparts. This finding is further supported by a population-based study, demonstrating an increased risk of abdominal obesity in young adult smokers [48]. Variations in the research methodology can likely explain these discrepancies. Differences in how key variables are defined and measured, the specific populations studied, the research design employed, and the inclusion of covariates in statistical models can all influence the comparability across studies. While the exact mechanisms remain unclear, smoking is known to be associated with behaviors that contribute to body weight gain, such as physical inactivity [49,50] and unhealthy dietary choices [51,52]. Additionally, changes in glucocorticoid metabolism triggered by smoking [53] and potential stress related to smoking cessation [54] might be contributing factors. Further research is needed to fully elucidate the causal pathways linking smoking and obesity.

According to our results, young adults with low physical activity levels were more than twice as likely to be overweight or obese compared to their more active counterparts. Regular physical activity is regarded as one of the most crucial lifestyle factors for the prevention and management of obesity [54,55,56,57]. The European Practical and Patients-Centered Guidelines for Obesity Management recommend a walking program of at least 30 min a day, five days a week, for overweight and obese adults. This recommendation can be further enhanced by incorporating resistance training exercises targeting major muscle groups twice a week [58,59]. Young people often reduce their participation in regular physical activity during their adolescent years, and this decline typically persists into the transition from adolescence to adulthood [60,61]. This lack of activity contributes significantly to the global health burden. According to the World Health Organization, over 1.4 billion adults, or 27.5% of the global adult population, are insufficiently active [62]. Research further highlights the long-term consequences of inactivity during adolescence. Physically inactive adolescents had a 3.9 times higher risk of developing overall obesity in adulthood, and even a 4.8 times higher risk of developing abdominal obesity [63]. Once obesity sets in, physical activity levels stay very low, resulting in low energy expenditure. This suggests that a sedentary lifestyle during adolescence can initiate obesity, creating a vicious circle, where reduced activity and low energy expenditure lead to further body weight gain. The low physical activity levels observed among university students have been considered a serious and worrying issue. Studies in Norway, for example, report that, regarding university students, less than a quarter of males and less than a fifth of females meet the recommended activity guidelines [64]. These findings underscore the importance of healthcare providers actively encouraging college students to increase their physical activity levels and, subsequently, improve their overall health and well-being.

Psychological stress is a significant concern for university students, negatively affecting academic performance, physical health, and mental well-being [29]. Research suggests that stress can trigger unhealthy dietary changes in both genders, with women exhibiting a preference for sweets and snacks while men tend to gravitate towards fast food and meat [65,66]. This stress-induced overeating can lead to uncontrolled calorie intake [67]. Interestingly, unhealthy dietary choices and skipping meals have also been linked to increased stress-related behaviors and symptoms in adolescents [68]. Sleep duration is another crucial factor, influencing both stress and obesity. A shorter sleep duration has been associated with a heightened risk of depression and anxiety, which could be related to stress-related behaviors [69]. Our study mirrored these findings, demonstrating that young adults experiencing moderate or high stress were more than twice as likely to be overweight or obese compared to their low-stress counterparts. Furthermore, another study reported a pre-obesity prevalence of 39.5% among college students, and that the risk of stress increased with established obesity [28]. These findings strongly suggest a link between stress and obesity in young adults. Therefore, two potential factors might explain the co-existence of obesity and stress, which are sleep duration and high-calorie intake. Studies have shown that a long-term high-fat diet can elevate serum corticosterone levels and hippocampal CYP11B1 expression, ultimately leading to increased HPA axis activity and anxiety [70]. Additionally, the gut microbiota impacts obesity-related metabolic factors, influencing anxiety-like behavior through the microbiota–gut–brain axis [71]. Interestingly, calorie restriction has been shown to have positive effects, including preventing brain atrophy, enhancing neurogenesis, and alleviating anxiety [72,73]. One study even observed that calorie restriction helped reduce obesity-associated anxiety in students, with a more significant improvement seen in obese participants compared to underweight ones [28].

Overweight and obesity in adults are reported to lead to impaired sleep [30,74,75]. A study examining over 1500 college students in the US and North Korea found that both short and long sleep durations increased the risk of obesity and overweight [30]. Similarly, our study found that young adults with inadequate sleep quality were 86% more likely to be overweight or obese compared to those with good sleep. The relationship between body weight status and sleep issues appears to be bidirectional. Lifestyle factors can significantly impact sleep quality, and there is a well-established connection between sleep and mental health among university students [76]. We must also consider other co-existing behaviors that can disrupt sleep, such as alcohol abuse, physical inactivity, depression, and anxiety [22,77,78]. These factors are also known to be associated with an increased risk of overweight and obesity. Furthermore, excess body weight can lead to breathing problems and sleep apnea, which can further worsen sleep issues [79,80]. Inflammatory markers, which are often elevated in obese individuals, may also play a significant role in disrupting sleep quality [81].

The currently available evidence about the association between overweight and obesity and the level of academic performance remains controversial. While some studies report no significant association or even a negative correlation [82,83,84,85], our results suggest a different perspective. Our study found that 57% of young adults with good academic performance had a higher prevalence of overweight or obesity compared to those with very good/excellent performance. This finding aligns with research by Aleidi et al. (2020) [86], who reported lower Grade Point Averages (GPAs) among students with higher BMI. There is also evidence supporting the negative impact of excess body weight on academic achievement. A study has suggested that obese female students may experience lower grades, diminished confidence, and decreased participation in courses [87]. Increased body weight might also lead to emotional and educational challenges, such as lower self-esteem and reduced academic engagement [88]. These issues could potentially stem from difficulties with eating regulation or body weight stigma. Another factor to consider is physical activity. Studies have shown that students with lower grades tend to have lower physical activity levels compared to their high-performing counterparts [46]. This suggests that physical activity might be a mediating factor in the weight–academic performance relationship. Students with high academic performance may be more likely to engage in regular exercise, leading to a healthier body weight and potentially improved cognitive function [89,90]. While some studies have found no significant associations, the potential negative impacts of excess body weight and the benefits of physical activity highlight the importance of further research in this area. Additionally, investigating the role of social and economic factors could provide valuable insights into these complex relationships.

A key strength of this study is that it was conducted in several Greek regions, encompassing both urban and rural settings. This enhances the generalizability of our findings and suggests that the sample is quite representative of young adults in Greece. Moreover, taking into consideration previous studies that showed that student nutritional habits and lifestyle, which are significantly associated with BMI status, are rather similar and common to most European countries, the findings of the present study may be representative of at least European countries [26,27]. Furthermore, the use of objective BMI measurements, rather than relying on self-reported data, ensured the accurate categorization of participants into overweight or obese categories, minimizing recall bias. Additionally, physical activity, stress, and sleep quality were evaluated by well-recognized and validated questionnaires through face-to-face interviews with qualified personnel, which may reduce some reporting bias. 

This study has also some limitations, which need to be considered. The cross-sectional design of the study limits our ability to definitively establish causal relationships between stress, sleep quality, and overweight/obesity. To gain a more comprehensive understanding of the links between stress, sleep quality, and obesity, future research could include participants from various educational stages, such as primary and secondary school students. Additionally, employing longitudinal designs would allow for the exploration of causal relationships between these factors in college students. This could provide valuable insights into the potential development of overweight and obesity, paving the way for preventative interventions. Furthermore, future research could explore the role of specific dietary patterns or investigate interventions aimed at improving sleep quality and managing stress in young adults. Moreover, it should be noted that the currently available experimental evolution studies do not involve several rounds of sociodemographics, academic performance, and physical activity. Thus, since IPAQ, PSS, and PSQI questionnaires were used in the present study, our results and their comparison with the existing relevant studies should be interpreted with caution.

## 5. Conclusions

This study highlights the complex interplay between stress, sleep quality, and overweight/obesity in young adults in Greece. Our findings have supported evidence for a significant association between these factors, indicating that young adults experiencing stress and poor sleep quality are more likely to be overweight or obese. These results underscore the importance of a multifaceted approach to body weight management in this population. University programs and initiatives can play a crucial role in promoting healthy lifestyles and well-being. Investing in holistic wellness programs that address stress, sleep, diet, and physical activity can empower young adults to make positive lifestyle choices that can promote healthy body weight management and overall well-being throughout their academic journey and beyond.

## Figures and Tables

**Table 1:** Descriptive statistics of the enrolled young adults.

Characteristics (*n* = 2116)	Descriptive Statistics
**Age (mean ± SD; years)**	21.4 ± 2.3
**Gender (*n*, %)**	
Male	1100 (48.8%)
Female	1016 (52.0%)
**Nationality (*n*, %)**	
Greek	1672 (79.0%)
Other	444 (21.0%)
**Type of residence (*n*, %)**	
Urban	1293 (61.1%)
Rural	823 (38.9%)
**Family economic status**	
Low	881 (41.6%)
Medium	830 (39.2%)
High	405 (19.1%)
**Living status (*n*, %)**	
Living with family	1008 (47.6%)
Living alone	1108 (52.3%)
**Parents marital status**	
No divorced	1332 (63.0%)
Divorced	784 (37.0%)
**Smoking status**	
No smokers	1312 (62.0%)
Smokers	804 (38.0%)
**Type of Studies**	
Biomedical studies	952 (45.0%)
Other studies	1164 (55.0%)
**Academic performance**	
Good	809 (38.2%)
Very good	961 (45.4%)
Excellent	346 (16.4%)
**Employment status**	
Employee	489 (23.1%)
No employee	1627 (76.9%)
**Physical activity (*n*, %)**	
Low	1024 (48.4%)
Medium	715 (33.8%)
High	377 (17.8%)
**BMI (*n*, %)**	
Normal weight	1597 (75.5%)
Overweight	336 (15.9%)
Obese	183 (8.6%)
**Perceived stress (*n*, %)**	
Low	965 (45.6%)
Moderate	739 (34.9%)
High	412 (19.5%)
**Sleep Quality (*n*, %)**	
Adequate	1322 (62.5%)
Inadequate	794 (37.5%)

**Table 2:** Associations of BMI status with sociodemographic, anthropometric, and lifestyle factors of the enrolled young adults.

Characteristics (*n* = 2116)	BMI Status			
	Normal Weight 1597 (75.5%)	Overweight 336 (15.9%)	Obesity 860 (8.6%)	*p*-Value
**Age (mean ± SD; years)**	21.4 ± 2.4	21.2 ± 2.3	21.1 ± 2.3	*p* = 0.1815
**Gender (*n*, %)**				*p* < 0.0001
Male	707 (44.3%)	194 (57.7%)	115 (62.8%)	
Female	890 (55.7%)	142 (42.3%)	68 (37.2%)	
**Nationality (*n*, %)**				*p* = 0.0491
Greek	1277 (80.0%)	263 (78.3%)	132 (72.1%)	
Other	320 (20.0%)	73 (21.7%)	51 (27.9%)	
**Type of residence (*n*, %)**				*p* < 0.0001
Urban	913 (57.2%)	240 (71.4%)	140 (76.5%)	
Rural	684 (42.8%)	96 (28.6%)	43 (23.5%)	
**Family economic status**				*p* = 0.0152
Low	643 (40.2%)	154 (45.8%)	84 (45.9%)	
Medium	600 (37.6%)	147 (43.8%)	83 (45.4%)	
High	354 (22.2%)	35 (10.4%)	16 (8.7%)	
**Living status (*n*, %)**				*p* = 0.0005
Living with family	886 (55.5%)	145 (43.1%)	77 (42.1%)	
Living alone	711 (44.5%)	191 (56.9%)	106 (57.9%)	
**Parents marital status**				*p* = 0.0309
No divorced	1026 (64.2%)	206 (61.3%)	100 (54.6%)	
Divorced	571 (35.8%)	130 (38.7%)	83 (45.4%)	
**Smoking status**				*p* < 0.0001
No smokers	1049 (65.7%)	184 (54.8%)	79 (43.2%)	
Regular smokers	548 (34.3%)	152 (45.2%)	104 (56.8%)	
**Type of Studies**				*p* = 0.7767
Biomedical studies	878 (55.0%)	189 (56.2%)	97 (53.0%)	
Other studies	719 (45.0%)	147 (43.8%)	86 (47.0%)	
**Academic performance**				*p* = 0.0002
Good	567 (35.5%)	144 (42.9%)	98 (53.5%)	
Very good	756 (47.3%)	143 (42.5%)	62 (33.9%)	
Excellent	274 (17.2%)	49 (14.6%)	23 (12.6%)	
**Employment status**				*p* = 0.4022
Employee	395 (24.7%)	45 (13.4%)	49 (26.8%)	
No employee	1202 (75.3%)	291 (86.6%)	134 (73.2%)	
**Physical activity (*n*, %)**				*p* < 0.0001
Low	711 (44.5%)	187 (55.6%)	126 (68.8%)	
Medium	545 (34.1%)	125 (37.2%)	45 (24.6%)	
High	341 (21.4%)	24 (7.1%)	42 (6.6%)	
**Perceived stress (*n*, %)**				*p* < 0.0001
Low	871 (54.5%)	69 (20.5%)	25 (13.7%)	
Moderate	516 (32.3%)	139 (41.4%)	84 (45.9%)	
High	210 (13.2%)	128 (38.1%)	74 (40.4%)	
**Sleep Quality (*n*, %)**				*p* < 0.0001
Adequate	1112 (69.6%)	137 (40.8%)	73 (39.9%)	
Inadequate	485 (30.4%)	199 (59.2%)	110 (60.1%)	

**Table 3:** Multivariate binary logistic regression analysis for BMI status of study population.

Characteristics	BMI Status (Overweight & Obesity vs. Normal Weight)	
	RR * (95% CI **)	*p*-Value
**Age** (Over/Below mean value)	1.12 (0.24–1.95)	*p* = 0.6701
**Gender** (Male/Female)	1.23 (0.61–1.88)	*p* = 0.1398
**Nationality** (Greek/Other)	0.95 (0.26–1.62)	*p* = 0.3879
**Type of residence** (Urban/Rural)	1.88 (1.56–2.27)	*p* = 0.0056
**Family economic status** (Low/Medium & High)	1.28 (0.69–1.91)	*p* = 0.3032
**Living status** (Living alone/Living with family)	1.09 (0.47–1.85)	*p* = 0.1594
**Parents marital status** (Divorced/No divorced)	1.05 (0.40–1.72)	*p* = 0.2701
**Smoking status** (Regular smokers/No smokers)	2.04 (1.79–2.28)	*p* = 0.0012
**Type of Studies** (Biomedical studies/Other studies)	0.95 (0.21–1.92)	*p* = 0.8877
**Academic performance** (Good/Very good & Excellent)	1.57 (0.97–2.12)	*p* = 0.0103
**Employment status** (Employee/No employee)	0.96 (0.28–1.74)	*p* = 0.6801
**Physical activity** (Low/Medium & High)	2.15 (1.87–2.41)	*p* = 0.0008
**Perceived stress** (Moderate & High/Low)	2.43 (2.19–2.64)	*p* = 0.0005
**Sleep quality** (Inadequate/Adequate)	1.86 (1.60–2.14)	*p* = 0.0007

* Relative Ratio: RR; ** CI: Confidence Interval.

## Data Availability

The data of the present study are available upon request to the corresponding author due to private policy.
